# Supplementary material for: Affinity Maturation and Light-Chain-Mediated Paratope Diversification Anticipate Viral Evolution
Source: bioRxiv. 2025 Aug 28:2025.08.27.672735. Preprint. [Version 1] doi: 10.1101/2025.08.27.672735 (PMC12407817; doi:10.1101/2025.08.27.672735)
Supplement: Supplement 1 [file NIHPP2025.08.27.672735v1-supplement-1.pdf]

Figure legends:

Supplemental Figure 1. Epitope groups defined by deep mutational scanning profiles (source data: Cao et al., 2023) and their distribution across IGHV genes. Pre-calculated escape scores are mapped onto RBD structures using a red color scale.

Supplemental Figure 2. Memory B cell sorting from an infection-naïve, Wuhan-mRNA vaccinee cohort. (A) Cohort summary. (B) Plasma reactivity to nucleocapsid protein from 9 study subjects, and the AUC ratio relative to blank (background control) was plotted. Nine positive control subjects (taken 1 month post-infection) were included. (C) Gating strategy for spike-reactive memory B cell sorting.

Supplemental Figure 3. Contact details comparing antibodies V30V4 and Omi3. (A) Atomic contact counts. (B) Representative interaction snapshots showing RBD mutations K417N, N501Y, and Y505H.

Supplemental Figure 4. Interface Comparisons between V30V4 and P5A-3A1, a fully-germline IGHV3-53:IGKV3-20 antibody. (A) **I**nteraction surface of Wuhan RBD with both V30V4 and P5A-3A1 (PDB ID: 7D0C) at positions of highly convergent IGHV3-53/66 SHM. (B) Pairwise breakdown of Wuhan RBD interacting positions and hallmark SHM sites on V30V4 and P5A-3A1.

Supplemental Figure 5. Neutralization Breadth and SHM comparisons between Cao IGHV3-53/66 antibodies by exposure group. (A) Proportion of antibodies in “Wuhan exposed” and “BTI” groups that neutralize all tested strains through the noted strain. (B) SHM counts along VH gene segments for Cao BTI antibodies. (C) SHM counts along VH gene segments for Cao Wuhan exposed antibodies.

Supplemental Figure 6. Noteworthy features of IGHV3-53/66 antibodies. (A) Structural comparison between V30V4, containing the highly enriched, neutralization-breadth-associated residue 115T, and P5A-3A1, containing the germline residue 115F. (B) Highlighted positions with differential mutational frequencies between Wuhan-mRNA vaccine derived antibodies from this study, and Cao BTI antibodies.

Supplemental Figure 7. Association between CDRH3 length and light chain pairing. (A) CDRH3 length breakdown for IGHV3-53/66 antibodies by exposure and neutralization breadth category. (B) Counts and statistics for IGHV3-53/66 antibodies with 11aa CDRH3s and IGKV1-9 pairing by exposure group. (C) Overall frequencies of the intersection of CDRH3 lengths and 3 common light chain pairings for IGHV3-53/66 antibodies by exposure group. Data from Cao dataset.

Supplemental Figure 8. Distance measurements describing positional gradient of IGHV3-53/66 antibodies by paired light chain. (A) Superimposition of all analyzed IGHV3-53/66 antibody structures paired with 3 common light chains. (B) Root mean squared distance (RMSD) calculations between average structures of light-chain defined IGHV3-53/66 antibody groups. Measurements are made across all of VL, across only the CDRL1 region, and across positions constituting conserved B-sheets. (C) Per-residue root mean squared fluctuation (RMSF) measurements across all analyzed IGHV3-53/66 antibody structures paired with 3 common light chains, along positions of conserved B-sheets.

Supplemental Figure 9. Light chain contact details. Comparing antibody structures paired with IGKV1-9, IGKV1-33, and IGKV3-20 at residues (A) 498, (B) 501, and (C) 505. (D) Average contact frequency per light chain group. In the network diagram, node size reflects the number of contributing PDB structures, while edge length is inversely proportional to the average contact count. Numerical values of average contacts are indicated on the connecting edges.

bioRxiv preprint doi: https://doi.org/10.1101/2025.08.27.672735; this version posted August 28, 2025. The copyright holder for this preprint (which was not certified by peer review) is the author/funder, who has granted bioRxiv a license to display the preprint in perpetuity. It is made available under aCC-BY 4.0 International license.

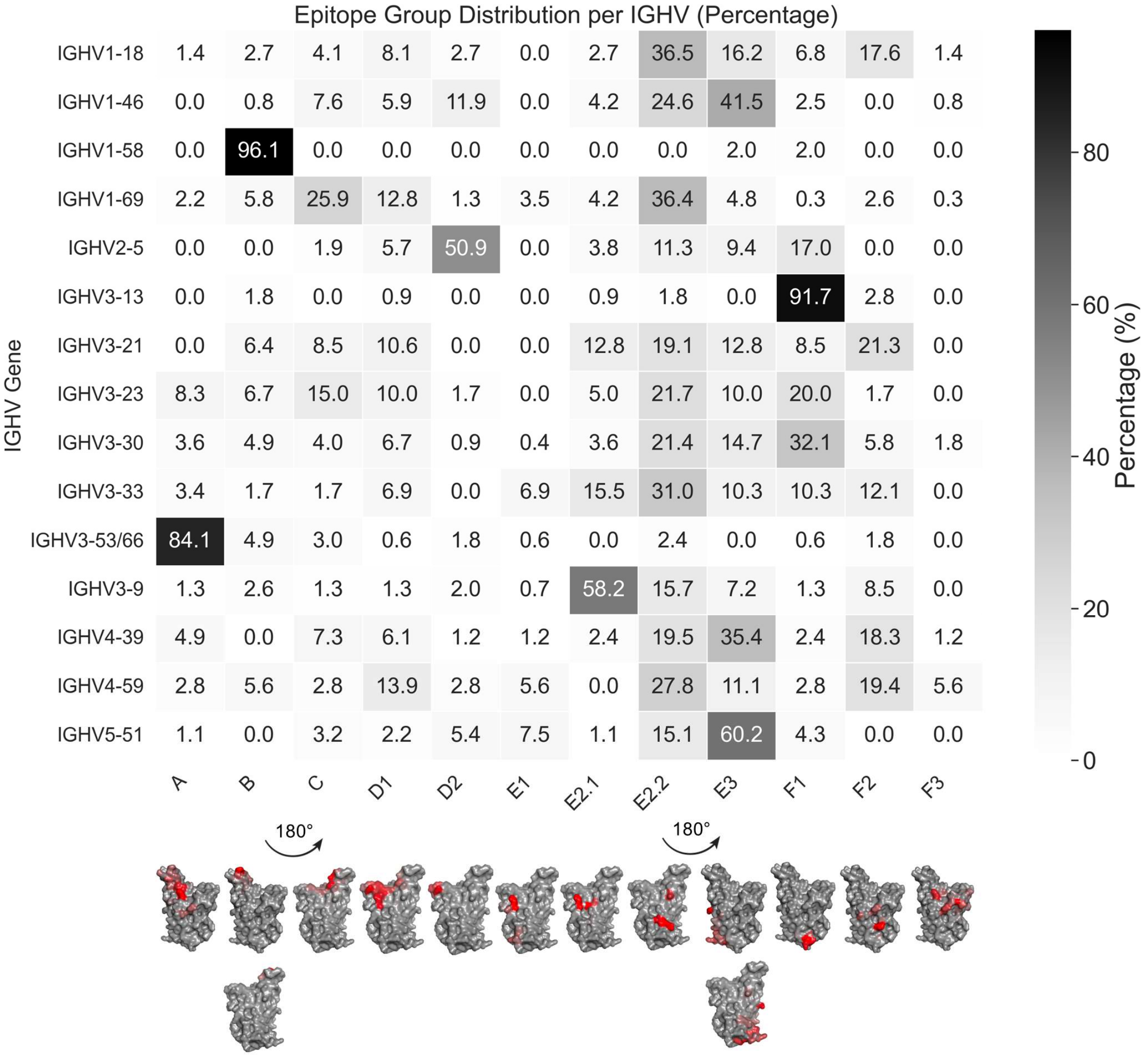

Supplemental Figure 1

bioRxiv preprint doi: https://doi.org/10.1101/2025.08.27.672735; this version posted August 28, 2025. The copyright holder for this preprint (which was not certified by peer review) is the author/funder, who has granted bioRxiv a license to display the preprint in perpetuity. It is made available under aCC-BY 4.0 International license.

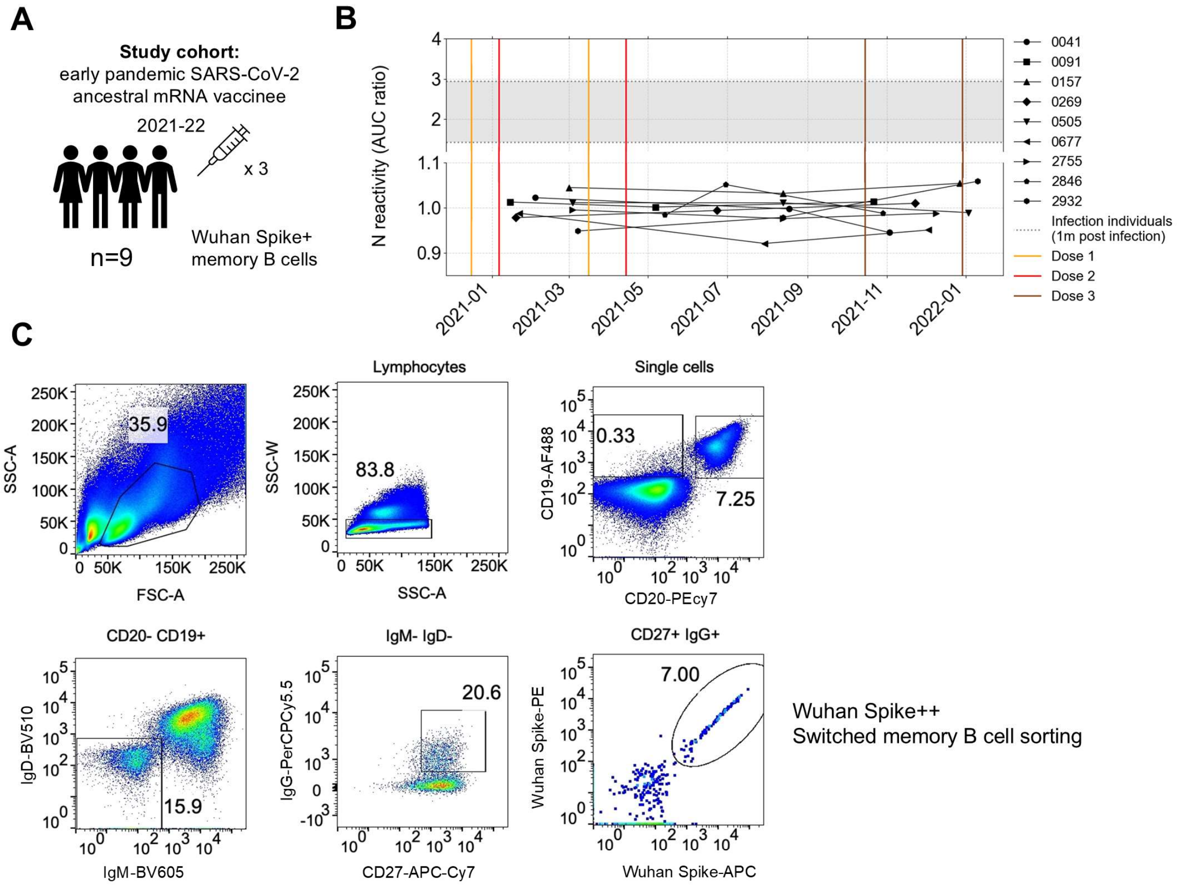

Supplemental Figure 2

bioRxiv preprint doi: https://doi.org/10.1101/2025.08.27.672735; this version posted August 28, 2025. The copyright holder for this preprint (which was not certified by peer review) is the author/funder, who has granted bioRxiv a license to display the preprint in perpetuity. It is made available under aCC-BY 4.0 International license.

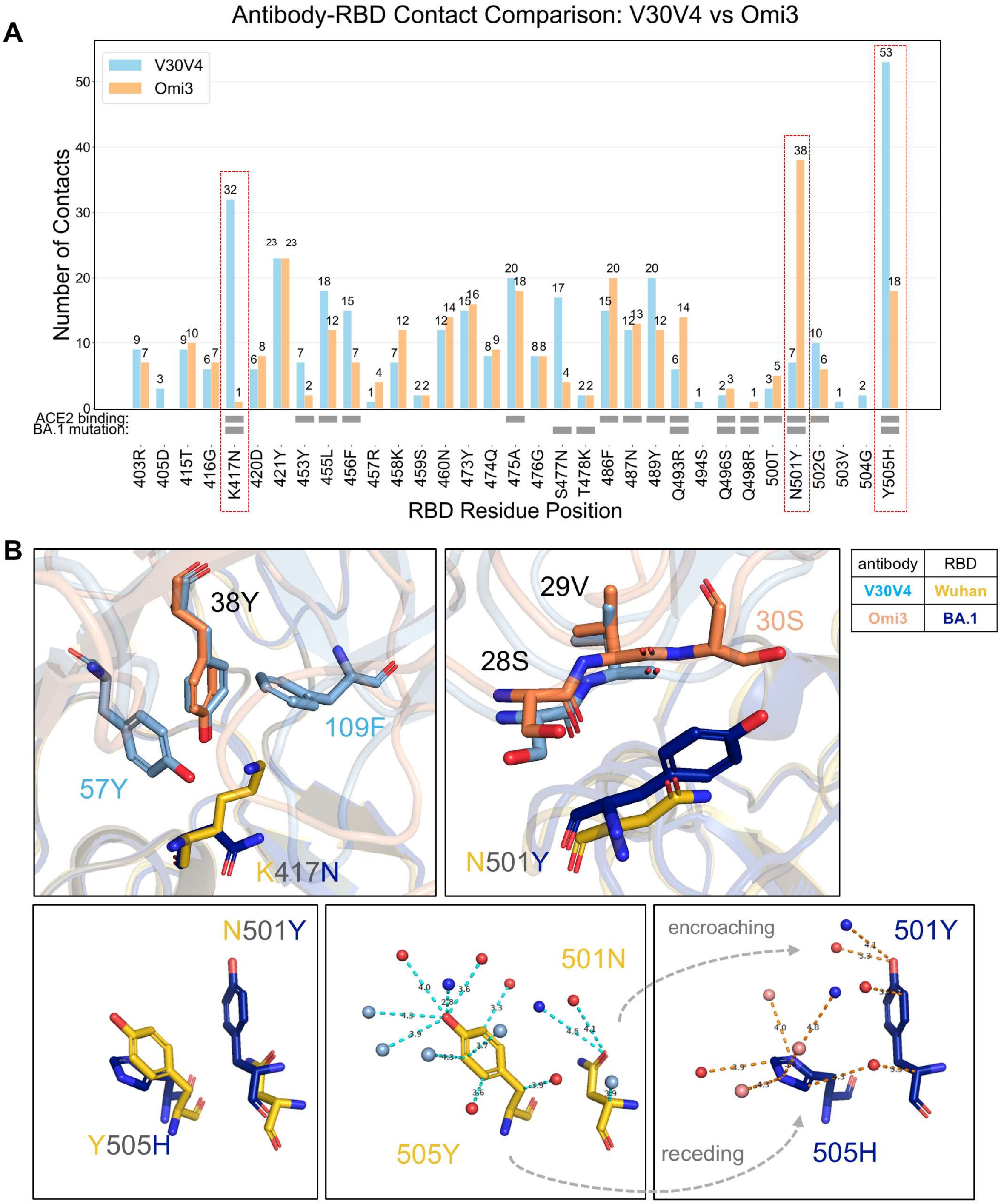

Supplemental Figure 3

bioRxiv preprint doi: https://doi.org/10.1101/2025.08.27.672735; this version posted August 28, 2025. The copyright holder for this preprint (which was not certified by peer review) is the author/funder, who has granted bioRxiv a license to display the preprint in perpetuity. It is made available under aCC-BY 4.0 International license.

A

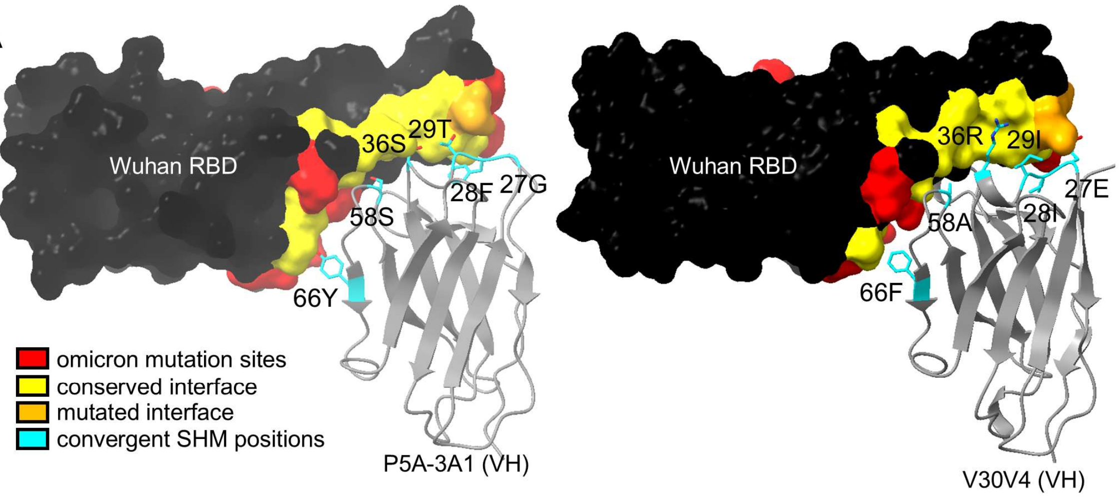

B

Pairwise Contacts

V30V4 (VH) — CDRH1 / CDRH2 — RBD pos #

27E	28I	29I	36R	58A	66F
476	487	475	458	421	415
477		476	473	457	416
478		477	474	458	
487			475	473	
			476		

7D0C (VH) — RBD pos #

27G	28F	29T	36S	58S	66Y
476	475	475	458	421	415
	477	476	473	457	416
	487	477	474	458	420
			475	473	

conserved interface
mutated interface

Supplemental Figure 4

bioRxiv preprint doi: https://doi.org/10.1101/2025.08.27.672735; this version posted August 28, 2025. The copyright holder for this preprint (which was not certified by peer review) is the author/funder, who has granted bioRxiv a license to display the preprint in perpetuity. It is made available under aCC-BY 4.0 International license.

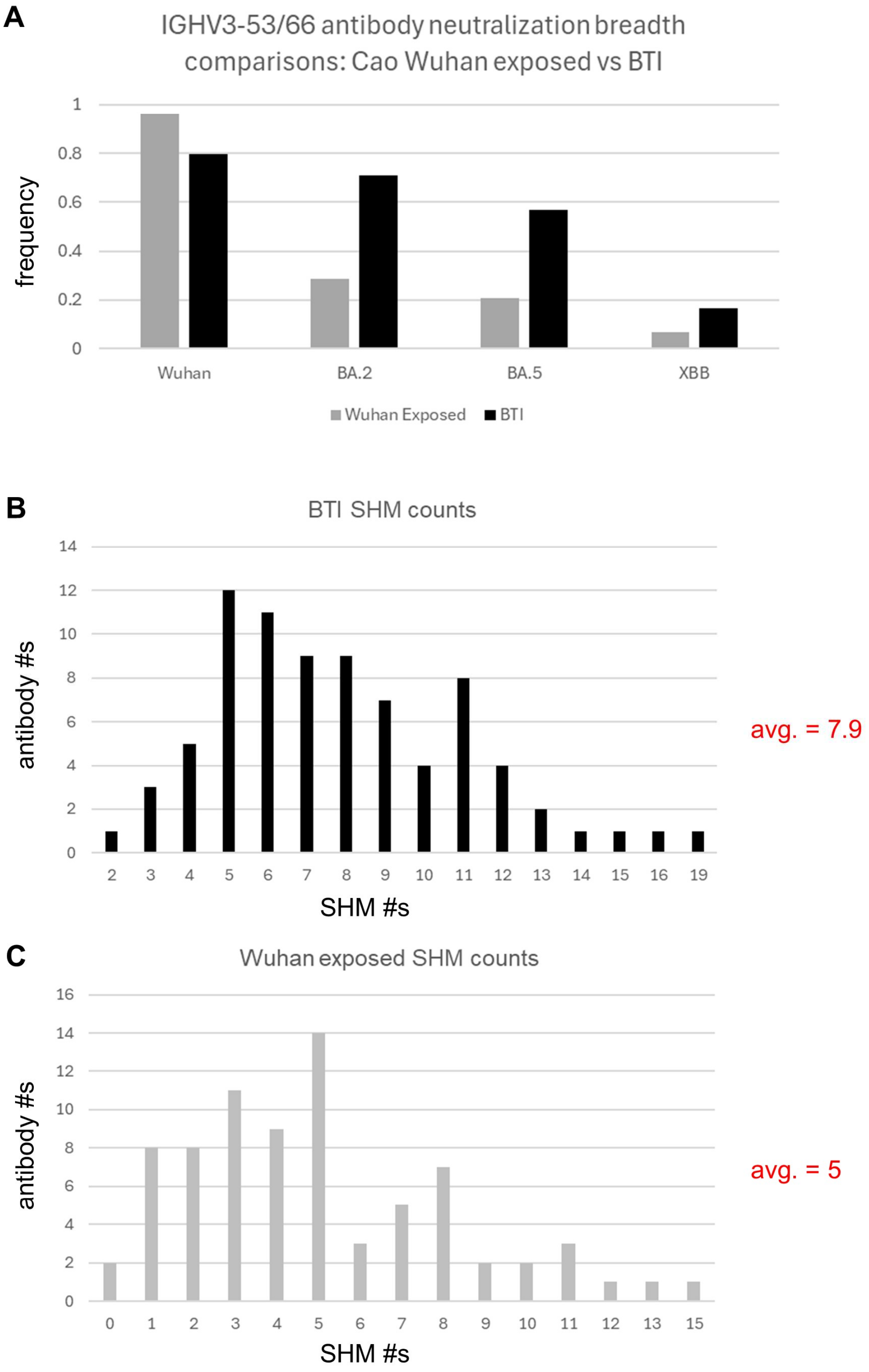

Supplemental Figure 5

bioRxiv preprint doi: https://doi.org/10.1101/2025.08.27.672735; this version posted August 28, 2025. The copyright holder for this preprint (which was not certified by peer review) is the author/funder, who has granted bioRxiv a license to display the preprint in perpetuity. It is made available under aCC-BY 4.0 International license.

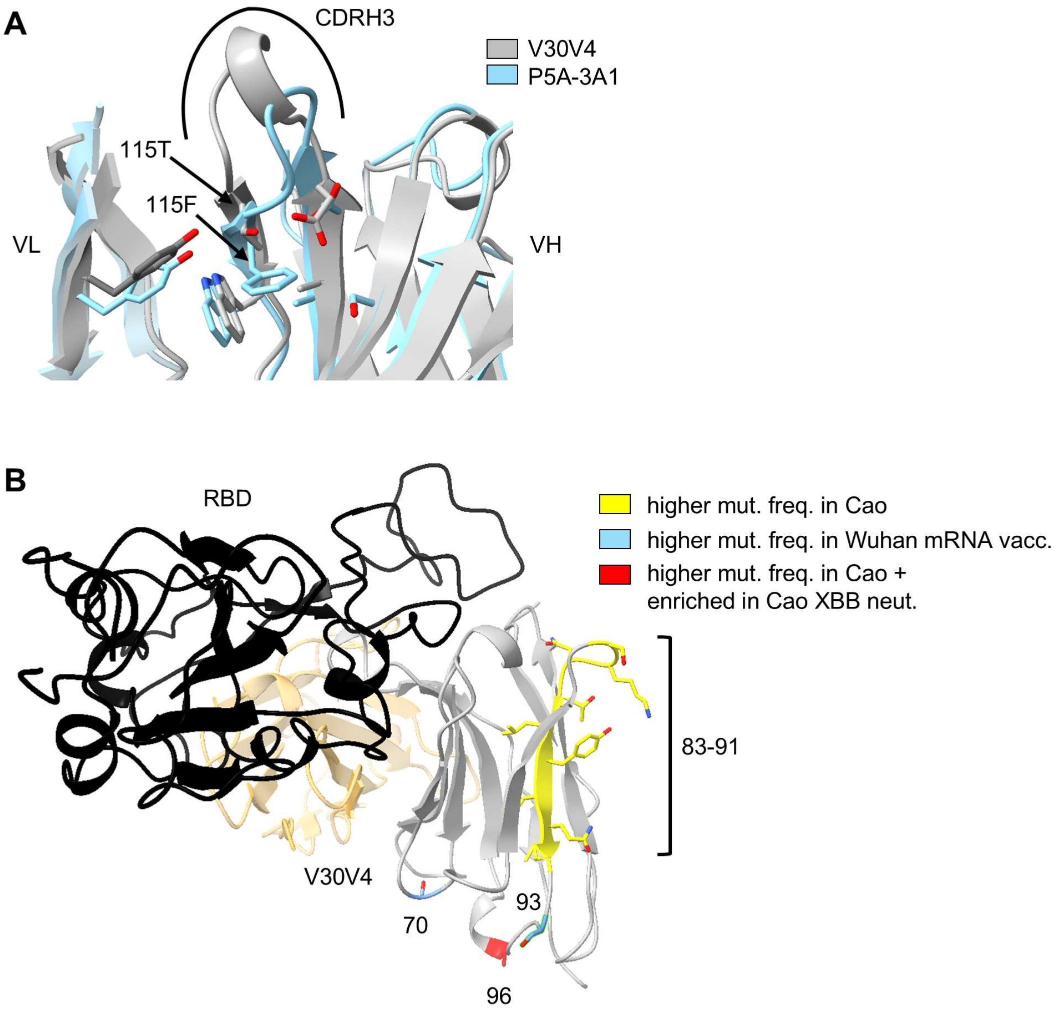

Supplemental Figure 6

bioRxiv preprint doi: https://doi.org/10.1101/2025.08.27.672735; this version posted August 28, 2025. The copyright holder for this preprint (which was not certified by peer review) is the author/funder, who has granted bioRxiv a license to display the preprint in perpetuity. It is made available under aCC-BY 4.0 International license.

A

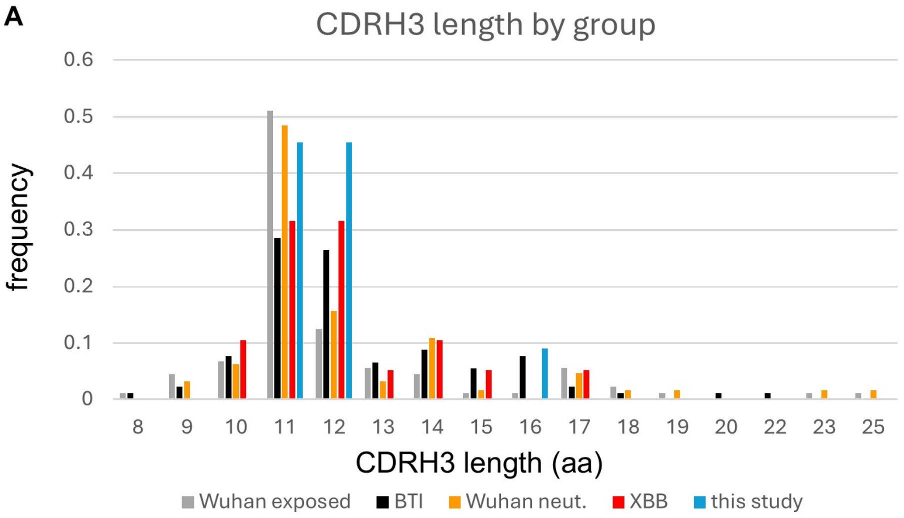

B

Wuhan Exposed

	KV1-9	not KV1-9	total
CDRH3 11	22	23	45
not 11	7	36	43
total	29	59	88

p val = 1.9E-3

phi = 0.347

BTI

	KV1-9	not KV1-9	total
CDRH3 11	11	15	26
not 11	5	60	65
total	16	75	91

p val = 9.0E-4

phi = 0.411

All Cao

	KV1-9	not KV1-9	total
CDRH3 11	33	38	71
not 11	12	96	108
total	45	134	179

p val = 1.5E-5

phi = 0.399

C

Wuhan Exposed

	KV1-9	KV1-33	KV3-20
CDRH3 11	0.759	0.200	0.667
CDRH3 12	0.069	0.050	0.25
other	0.172	0.750	0.0833

BTI

	KV1-9	KV1-33	KV3-20
CDRH3 11	0.688	0.280	0.231
CDRH3 12	0.063	0.160	0.615
other	0.250	0.560	0.154

Supplemental Figure 7

bioRxiv preprint doi: https://doi.org/10.1101/2025.08.27.672735; this version posted August 28, 2025. The copyright holder for this preprint (which was not certified by peer review) is the author/funder, who has granted bioRxiv a license to display the preprint in perpetuity. It is made available under aCC-BY 4.0 International license.

A

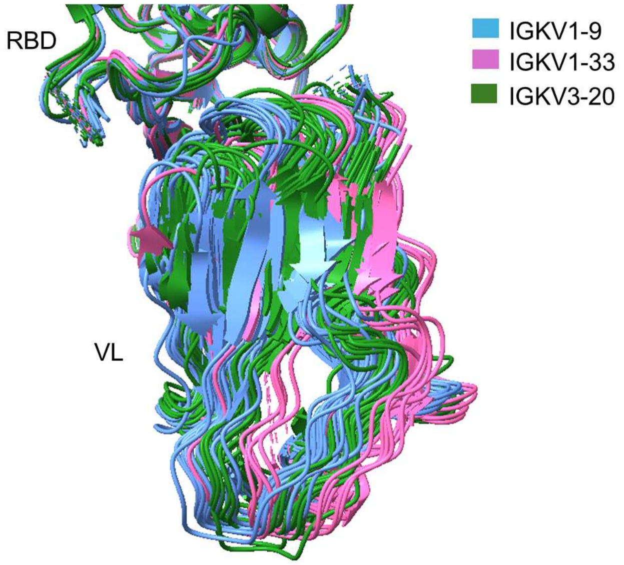

B

Between-Group RMSDs (Å)

Whole VL

	K1-9	K1-33	K3-20
**K1-9**		3.09	1.56
**K1-33**			1.98
**K3-20**			

CDRL1

	K1-9	K1-33	K3-20
**K1-9**		3.17	2.17
**K1-33**			2.08
**K3-20**			

B-sheets

	K1-9	K1-33	K3-20
**K1-9**		3.01	1.58
**K1-33**			1.67
**K3-20**			

C

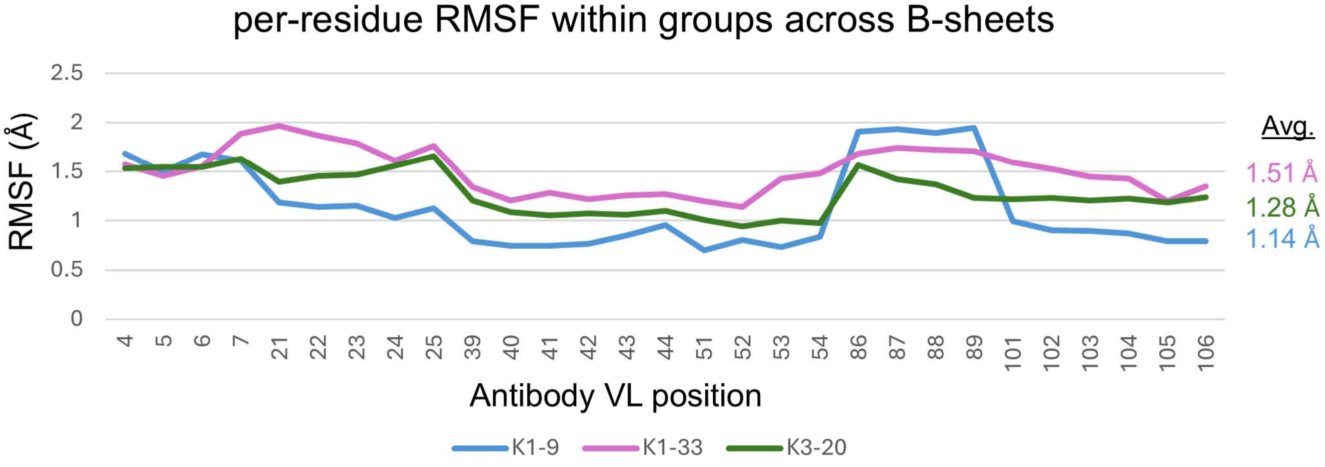

Supplemental Figure 8

bioRxiv preprint doi: https://doi.org/10.1101/2025.08.27.672735; this version posted August 28, 2025. The copyright holder for this preprint (which was not certified by peer review) is the author/funder, who has granted bioRxiv a license to display the preprint in perpetuity. It is made available under aCC-BY 4.0 International license.

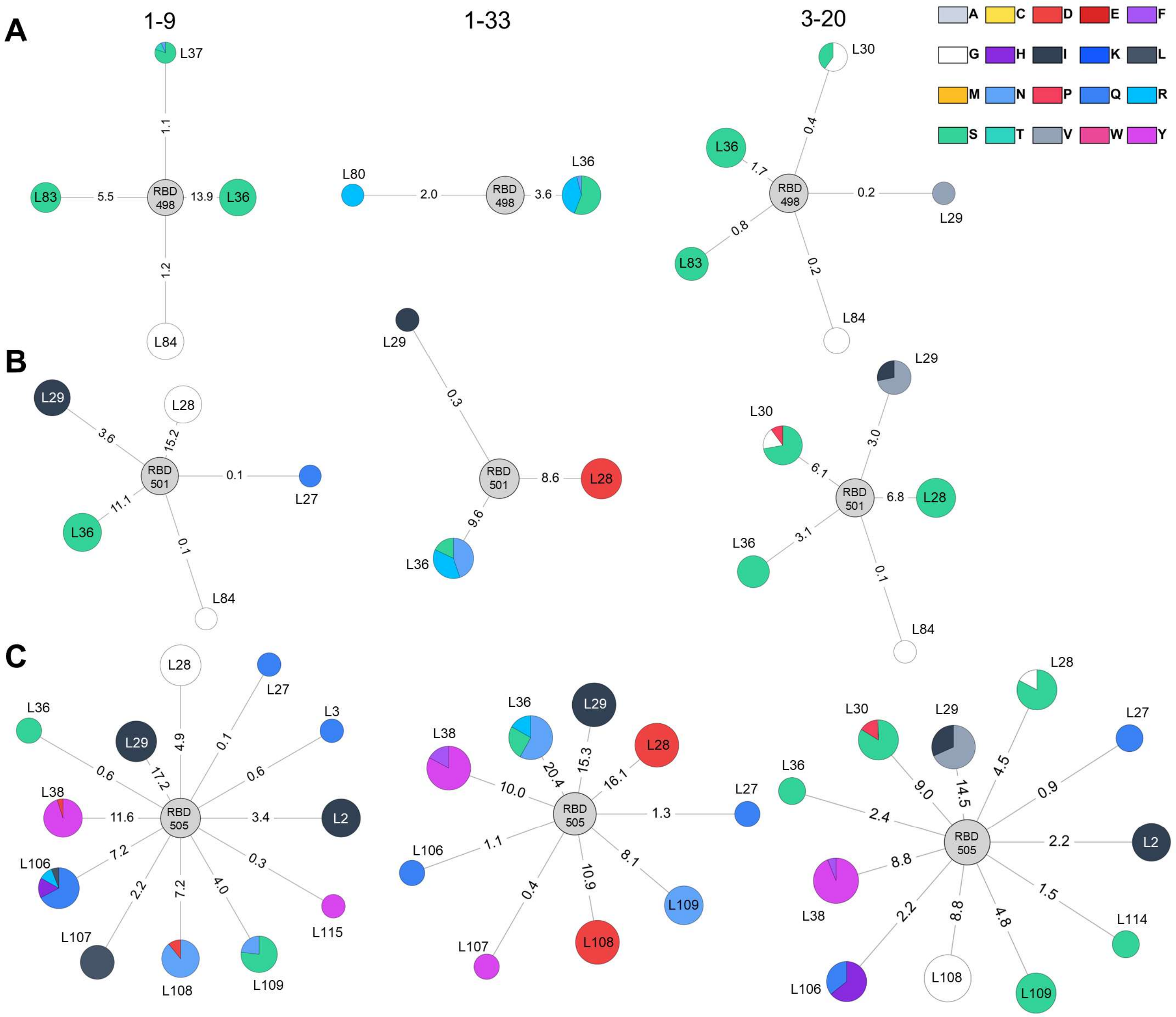

D

Light_Chain	RBD_Residues	Num_Structures	Avg_Total_Contacts	Avg_VH_Contacts	Avg_VL_Contacts
IGKV1-9	498	14	13.21	0	13.21
IGKV1-9	501	14	18.5	0	18.5
IGKV1-9	505	14	30.93	0	30.93
IGKV1-33	498	7	2.86	0	2.86
IGKV1-33	501	7	10.14	0	10.14
IGKV1-33	505	7	46.86	0	46.86
IGKV3-20	498	13	1.31	0	1.31
IGKV3-20	501	13	10.15	0	10.15
IGKV3-20	505	13	35.62	0	35.62

Supplemental Figure 9
